# Benchmarking ChatGPT and Other Large Language Models for Personalized Stage-Specific Dietary Recommendations in Chronic Kidney Disease

**DOI:** 10.3390/jcm14228033

**Published:** 2025-11-12

**Authors:** Makpal Kairat, Gulnoza Adilmetova, Ilvira Ibraimova, Abduzhappar Gaipov, Huseyin Atakan Varol, Mei-Yen Chan

**Affiliations:** 1 Department of Biomedical Sciences, School of Medicine, Nazarbayev University, Astana 010000, Kazakhstan; 2 Department of Medicine, School of Medicine, Nazarbayev University, Astana 010000, Kazakhstan; 3 Institute of Smart Systems and Artificial Intelligence, Nazarbayev University, Astana 010000, Kazakhstan

**Keywords:** renal health, dialysis, Artificial Intelligence (AI), ChatGPT, LLM, AI-assisted, dietary guidance

## Abstract

**Background**: Chronic kidney disease (CKD) requires strict dietary management tailored to disease stage and individual needs. Recent advances in artificial intelligence (AI) have introduced chatbot-based tools capable of generating dietary recommendations. However, their accuracy, personalization, and practical applicability in clinical nutrition remain largely unvalidated, particularly in non-Western settings. **Methods**: Simulated patient profiles representing each CKD stage were developed and used to prompt GPT-4 (OpenAI), Gemini (Google), and Copilot (Microsoft) with the same request for meal planning. AI-generated diets were evaluated by three physicians using a 5-point Likert scale across three criteria: personalization, consistency with guidelines, practicality, and availability. Descriptive statistics, Kruskal–Wallis tests, and Dunn’s post hoc tests were performed to compare model performance. Nutritional analysis of four meal plans (Initial, GPT-4, Gemini, and Copilot) was conducted using both GPT-4 estimates and manual calculations validated against clinical dietary sources. **Results**: Scores for personalization and consistency were significantly higher for Gemini and GPT-4 compared with Copilot, with no significant differences between Gemini and GPT-4 (*p* = 0.0001 and *p* = 0.0002, respectively). Practicality showed marginal significance, with GPT-4 slightly outperforming Gemini (*p* = 0.0476). Nutritional component analysis revealed discrepancies between GPT-4’s internal estimations and manual values, with occasional deviations from clinical guidelines, most notably for sodium and potassium, and moderate overestimation for phosphorus. **Conclusions**: While AI chatbots show promise in delivering dietary guidance for CKD patients, with Gemini demonstrating the strongest performance, further development, clinical validation, and testing with real patient data are needed before AI-driven tools can be fully integrated into patient-centered CKD nutritional care.

## 1. Introduction

Chronic kidney disease (CKD) is a significant global health burden, as it affects approximately 850 million people, which is 10% of the world’s population [1]. It is characterized as lasting damage in kidney structure and loss of its functions over time [2]. According to the current evidence-based guidelines, CKD is diagnosed in case of either kidney damage or decreased glomerular filtration rate (below 60 mL/min per 1.73 m^2^) that persists for a duration of three months or longer [3]. The main causes of CKD are non-communicable diseases like diabetes and hypertension [3,4]. Moreover, social factors like income level, education, access to healthcare, and overall living conditions have an influence on the incidence and progression of CKD. Unlike other non-communicable diseases, mortality related to CKD has been increasing steadily, making it the third fastest growing cause of death around the globe [5]. In Kazakhstan, around 1.7 million cases of CKD and 1485 deaths were reported by the year 2020 showing its significant burden [6].

While genetic factors do have an impact on disease onset and progression, most CKD cases are associated with nutritional factors and often preventable [7]. Proper nutrition and successful pharmacological treatment are some of the key factors that have a fundamental role in CKD management, which can maintain stable renal function and prevent complications to other organs [8]. According to Chen et al., special diets are required to maintain the special needs of CKD patients to achieve electrolyte balance, control fluid intake, and modify protein intake [9]. For example, excessive intake of sodium will lead to hypertension and water retention, while imbalances in potassium and phosphorus will lead to life-threatening complications like cardiac arrhythmias and bone diseases [9]. Protein intake also must be strictly regulated to prevent the accumulation of uremic toxins without causing protein–energy wasting. However, high-protein diets, frequently promoted in popular media for their ability to boost satiety and decrease fat mass, are becoming increasingly favored as a weight-loss approach [10]. Yet the trend has prompted concern about its potential ill effect on kidney function. Dietary management of CKD often presents severe challenges to patients, as they require highly individualized diet plans and face issues like lack of awareness of the constraints, dietary and cultural limitations, psychological challenges like stress or depression, and costs or inaccessibility of recommended foods and limited access to professional nutrition counseling [11,12].

In the past few years, AI has developed at an incredible speed and has been introducing many possibilities, like processing and analyzing huge amounts of medical data, early detection of diseases, creating personalized treatment recommendations, and enhanced patient engagement by providing companionship for elderly people in nursing homes in the healthcare sector [13,14]. In the field of nutrition, AI technologies have progressed to tackle several areas of dietary analysis, planning, and personalized nutritional interventions; the creation of smarter dietary analysis instruments; dietary identification and tracking technologies; and disease prevention and nutritional status modeling [15]. These developments have increased the precision, range, and customization of nutritional advice, addressing the individual’s specific health condition, metabolic condition, and cultural food needs.

Personalized nutrition is one of the emerging applications of AI, which delivers customized diet suggestions based on the health condition of the person, for instance, nutritional requirements and preferences and lifestyle. Natural Language Processing (NLP) and Machine Learning (ML) have been crucial in facilitating the ability to create personalized nutrition recommendations using actual-world data. NLP-based systems, such as ChatGPT and other conversational AI systems, have transformed medical literature, electronic health records, patient-created diet records to provide accurate diagnosis, and is used as an “internet dietician” that gives personalized diets and considers the patient data and preferences [16,17,18,19]. Adaptive meal suggestions are generated by these AI systems from a wide array of data points including expert advice, genetic susceptibility, biomarkers, and medical history [16]. By using big data, the systems try to provide nutritional health, prevent diet disorders, and assist individuals with certain nutritional requirements, such as individuals with chronic diseases [20].

Despite these developments, the effectiveness of AI-based nutrition tools is questionable, especially in managing complicated medical conditions such as noncommunicable diseases (NCDs). The review of early applications of LLMs in nutrition counseling highlighted their potential in recipe generation and dietary education, as well as their shortcomings inaccuracy in nutrition estimation and patient-specific tailoring issues [21]. Recent studies have investigated the effects of AI chatbots such as ChatGPT in designing meal plans and dietary advice. Papastratis et al. evaluated the ability of ChatGPT in designing personalized meal plans for individuals suffering from obesity, cardiovascular disease (CVD), and type 2 diabetes (T2D) [17]. The study contrasted ChatGPT recommendations with an evidence-based diet plan and concluded that while the AI plans were uniquely generated to user profiles, they required expert assessment to qualify as adequate and in conformity with clinical nutrition guidelines [17]. A supporting study by the same author reported a deep generative AI model, combining ChatGPT and a variational autoencoder, which maps user data into a meaningful latent space, to increase the accuracy of dietary advice. This system demonstrated encouraging performance in meal plans to regular nutrition recommendations matching and meal variety optimization [22]. The effectiveness of ChatGPT in producing dietary suggestions for certain NCDs was also measured by Ponzo et al. [23]. According to them, suggestions produced by ChatGPT were clear and followed guidelines in 55.5% to 73.3% of cases, depending on the condition [23]. In some cases, inconsistencies and contradictions were also found when ChatGPT had to deal with several overlapping conditions, which means that AI-produced dietary advice is not a substitute for professional nutritionists yet [23]. Further research has explored ChatGPT’s application to support specific patient groups. Wang et al. tested the application of ChatGPT in generating dialysis patients’ meal plans and reported extremely irregular estimates of nutrients, with the AI-generated plans having lower estimates of protein, fat, and sodium, all key nutrients [18]. These findings emphasize the need for strict validation of AI tools before their integration into clinical nutrition practice [18]. Similarly, Lo et al. researched multimodal ChatGPT application in diet evaluation and determined that GPT-4V was feasible for food detection and portion sizing [24]. Fine-tuning was, however, required for accurate nutrient calculation, particularly in real-case research [24].

Studies have confirmed that while ChatGPT-4 is doing better in the detection of certain nutrients such as potassium, it is still subpar in others, such as phosphorus [25]. Similarly, its whole application as a virtual nutritionist is promising in whole nutrition education but subpar in clinic application due to a lack of individualization and evidence-based completeness [16]. Therefore, AI suggestions must be validated for accuracy, personalization, and practicability in the clinic by adopting evidence-based nutrition treatment and patient individualization needs [26]. Further, data privacy issues, the pervasiveness of AI-powered bias, and the absence of real-time physiologic feedback complicate the use of such systems in the clinic [27]. Although AI can have the ability to deliver personalized care, its launch in the clinic needs to be well tested, controlled, and in conjunction with clinicians and tech suppliers.

AI has been used in CKD management as well, with a growing number of studies exploring its role in generating dietary recommendations. Bingöl et al. [28] conducted comparative evaluation study where the accuracy of LLMs such as GPT4, Gemini, Llama and a retrieval-augmented generation (RAG) model was evaluated by asking 12 English prompts based on the NKF-KDOQI 2020 guidelines. The study used 12 English prompts prepared for two CKD groups: non-dialysis CKD stages 3–5 and dialysis patients and they aimed to generate responses related to general dietary principles such as energy, protein, sodium, potassium, and phosphorus intake. The AI responses were assessed using 5-point Likert Scale by experts in chronic diseases nutrition field. While Bingöl et al. reported insights on the accuracy levels of these LLM models, they did not assess the full meal plans, personalization of responses and did not perform any nutritional analysis [28]. Similarly, Qarajeh et al. performed multimodal comparison of ChatGPT, Bard AI and Bing Chat to identify potassium and phosphorus content of 240 food items and classify them as low and high [25]. Even though Qarajeh et al. explored the accuracy level of different AI chatbots, they did not test the ability of AIs models to generate stage specific and comprehensive diet plan [25]. Despite the interest in AI driven dietary management of CKD, current research has been focusing primarily on Western populations. One of the gaps is that most of the AI based dietary platforms rely on Western nutritional standards which makes it unclear whether these models provide practical, culturally appropriate guidance for populations with distinct dietary traditions. Meanwhile, recent research by Adilmetova et al. investigate effectiveness of AI-driven dietary recommendations in Central Asian context, especially in Kazakhstan [29]. They utilized 50 culturally tailored mock patient scenarios to assess ChatGPT 4’s capacity to offer personalized, evidence-based dietary recommendations in English, Russian, and Kazakh [29]. The chatbot gave reasonable outputs, the responses in Kazakh language were much less readable and there was an issue with the representation of language and cultural suitability [29]. This study shows that dietary suggestions powered by AI in Central Asia are not well investigated. Authors emphasize the importance of creating locally adapted AI tools that will take into account regional dietary habits and linguistic diversity. Considering that Central Asian traditional diets are rich in dairy products, meat, and processed food, CKD management might be more challenging in these regions [30]. In these terms, AI can be used to provide diet recommendations with more organic meals that meet the CKD nutritional needs and help to control the consumption of potassium and phosphorus [31]. Moreover, it might help to increase the fiber consumption which is often overlooked part of Central Asian diet. Dietary adherence may be improved by customized meal plans that increase fiber intake without exacerbating CKD related problems [32]. Even though the study by Adilmetova et al. covered Central Asian cuisine, they did not focus on nutritional adequacy or stage specific differentiation of CKD cases leaving a gap within this field. Overall, all these research studies reveal that while dietary suggestion systems using AI hold a great promise, there remain unfulfilled areas regarding their precision, reliability, and utility in the context of clinical nutrition [21].

Our current research seeks to fill this gap by assessing AI-derived dietary recommendations for CKD patients in the context of Kazakhstan’s dietary culture. The main goal of this study is to evaluate how accurate, reliable, and personalized AI chatbots are in giving diet recommendations for people with CKD. In contrast to earlier studies, our study will require AI models to generate stage-specific, comprehensive diet plans with following analysis of their nutritional content. It also focuses on how practical and culturally suitable these diets are for people in Central Asia, where eating habits and available food options are different from those in Western countries. By assessing feasibility, accuracy, and potential adherence, this research will provide insight into the appropriateness of AI nutrition tools for diverse populations. Findings will determine if AI-generated diet recommendations can suitably respond to the local diet patterns, availability of food, and economic restrictions. The study will open doors to the development of culturally relevant AI-based dietary interventions to enhance CKD care in Central Asia.

## 2. Materials and Methods

### 2.1. Study Design

The present research applies quantitative, comparative analytic approaches to critically evaluate the dietary counsel provided by three AI-driven chatbots: ChatGPT (GPT-4, OpenAI, San Francisco, CA, USA), Gemini 2.0 (Google, Mountain View, CA, USA), and Copilot 365 (Smart mode, GPT-4, Microsoft, Redmond, WA, USA), to CKD patients across stages 1 to 5. These models were accessed on 10 February 2025 through their public web interfaces. These platforms did not provide detailed version numbers or allow control over generative parameters (e.g., temperature, seed), so all responses were generated with default settings. Evaluation consists of assessing quality, accuracy, and suitability of dietary counsel drawn from AI following important criteria framed by medical practitioners.

The research consists of three main parts:

1. AI-Based Diet Recommendation Generation—Obtaining dietary advice from GPT-4, Gemini, and Copilot for patients suffering from CKD at different stages of the disease.
2. Professional Evaluation of AI Recommendations—Clinicians compare AI-made recommendations to predetermined review standards.
3. Nutritional Comparison and Statistical Analysis—Comparison of nutritional content between AI-recommended diets and comparison of performance among different AI models by statistical analysis.

**Figure 1:**
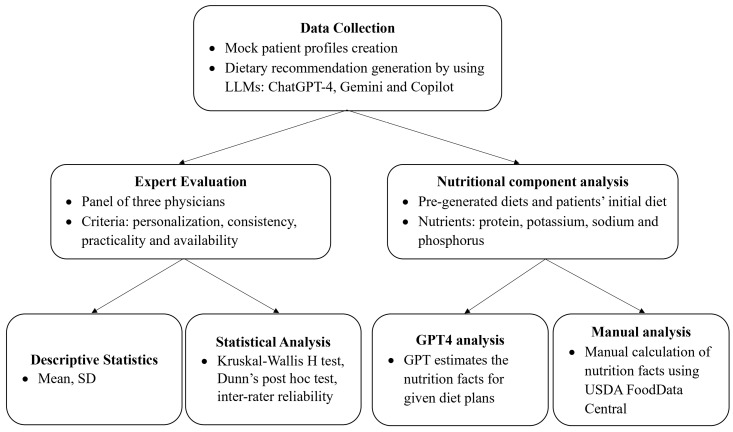
Overall Methodological Framework of the Study.

The overall methodological framework of the study is summarized in Figure 1, outlining the sequential process from AI-generated dietary recommendation collection to expert evaluation, statistical analysis, and nutritional component assessment.

### 2.2. Data Collection

#### 2.2.1. AI-Generated Dietary Recommendations

To assess the performance of AI chatbots, a series of standardized patient profiles were developed, representing CKD stages 1 to 5 (Appendix A). The profiles were authored by a postgraduate trainee in public health and a licensed physician in residency training, and then clinically validated by two additional resident physicians and a registered clinical dietitian. This process ensured medical accuracy and dietary relevance through collaborative review by practicing resident physicians and a clinical dietitian. These profiles include essential clinical and demographic variables such as age, gender, body mass index (BMI), dietary preferences, and relevant laboratory values (e.g., serum creatinine, potassium, phosphorus, sodium levels). Each AI model was tested using the same standardized prompt to ensure consistency. The prompt:

*“Provide a culturally appropriate, stage-specific dietary plan (breakfast, lunch, and dinner) for this patient with chronic kidney disease (CKD). Consider dietary restrictions (e.g., sodium, potassium, phosphorus, and protein intake) and incorporate foods commonly consumed in Central Asia.”*

was applied to all models. This approach ensured that they worked on the same task, allowing for a direct comparison of their adherence to clinical guidelines, practicality, and alignment with CKD dietary restrictions. Each model was queried once for each CKD case, generating a full-day meal plan that included breakfast, lunch, and dinner options. Breakfast, lunch, and dinner were evaluated separately for each case by three independent evaluators, and the resulting scores were averaged for analysis.

#### 2.2.2. Expert Evaluation of AI Recommendations

The quality of AI-generated dietary recommendations were assessed by a panel of independent three physicians specializing in CKD management. The evaluation framework is adapted from Adilmetova et al., employing a structured rubric to systematically assess the recommendations based on three key criteria: personalization, consistency, and practicality and availability [29]. These criteria assess how well AI-generated recommendations are tailored to individual needs (personalization), their alignment with evidence-based guidelines (consistency), and can be realistically implemented with locally accessible foods (practicality and availability).

Each recommendation was independently assessed using a 5-point Likert scale, where 1 represents poor adherence to the criterion and 5 indicates an excellent level of compliance. Breakfast, lunch, and dinner plans were evaluated separately for each model and criterion, with a total of 45 ratings (*n* = 45) per model–criterion–meal combination. The final scores will be averaged across evaluators to facilitate a comparative analysis of AI models. This evaluation approach ensures a standardized, evidence-based assessment of AI-generated dietary guidance for CKD patients. The detailed evaluation metrics and scales adapted from prior research are presented in Table A1 (Appendix B).

The use of the Likert scale is justified based on prior research evaluating AI-generated dietary recommendations. Recent studies have applied Likert-based frameworks to assess aspects such as accuracy, completeness, appropriateness, and clinical alignment. For instance, Ponzo et al. used a 6-point scale to evaluate ChatGPT’s adherence to KDIGO and KDOQI guidelines [23]. Similarly, Kim et al.employed a 0–10 scale to assess effectiveness, applicability, and flexibility of AI-generated diet plans [33]. A comprehensive summary of Likert-based evaluation approaches from multiple studies is presented in Table A2 (Appendix B). The adoption of a Likert scale ensures a structured, reliable, and quantifiable evaluation method, facilitating consistent comparison of AI-generated dietary recommendations across different models.

### 2.3. Data Analysis

All statistical analyses of AI generated dietary recommendations for CKD patients were performed using Stata/MP 18 software. The evaluation of AI-generated dietary recommendations was conducted through descriptive and inferential statistical methods, inter-rater agreement analysis, and nutritional component assessment.

#### 2.3.1. Descriptive Statistics

To summarize the overall performance of AI models (ChatGPT-4, Gemini, and Copilot), descriptive statistics were calculated for each evaluation category (personalization, consistency, practicality and availability) across all AI models. The results are expressed as median and interquartile range (IQR), mean ± standard deviation (SD), minimum and maximum values, and total number of ratings (*n*) for each model-criterion combination.

#### 2.3.2. Comparison of AI Models

To assess potential differences in quality of dietary recommendations across the three AI models, a Kruskal-Wallis test was performed. Given the ordinal nature of the evaluation score and the small sample size, this non-parametric test was selected. The Kruskal-Wallis test was used to determine whether there were statistically significant differences in expert ratings for personalization, consistency, and practicality across the three AI systems. This test does not assume normality and is appropriate for comparing medians between more than two independent groups. If statistically significant differences were detected (*p* < 0.05), a post-hoc Dunn’s test was conducted to determine whether there are specific pairwise differences between the AI models. In addition, Cliff’s delta (δ) was calculated for all pairwise model comparisons across personalization, consistency, and practicality to quantify both the strength and direction of differences between models [34]. Cliff’s delta (δ), a non-parametric effect size suitable for ordinal data, was interpreted using established benchmarks, where negligible (<0.15], small (0.15–0.33], medium (0.33–0.47], and large (0.47<) values were defined [34].

#### 2.3.3. Inter-Rater Agreement

To assess the consistency of evaluation by different reviewers, inter-rater reliability was quantified using Krippendorff’s Alpha. These reliability measures establish that the AI-recommended suggestions were rated uniformly across reviewers. The strength of agreement was interpreted using the classification by Landis and Koch [35]:

- 0.00–0.20 = slight agreement
- 0.21–0.40 = fair agreement
- 0.41–0.60 = moderate agreement
- 0.61–0.80 = substantial agreement
- 0.81–1.00 = almost perfect agreement.

#### 2.3.4. Nutritional Component Analysis

**Figure 2:**
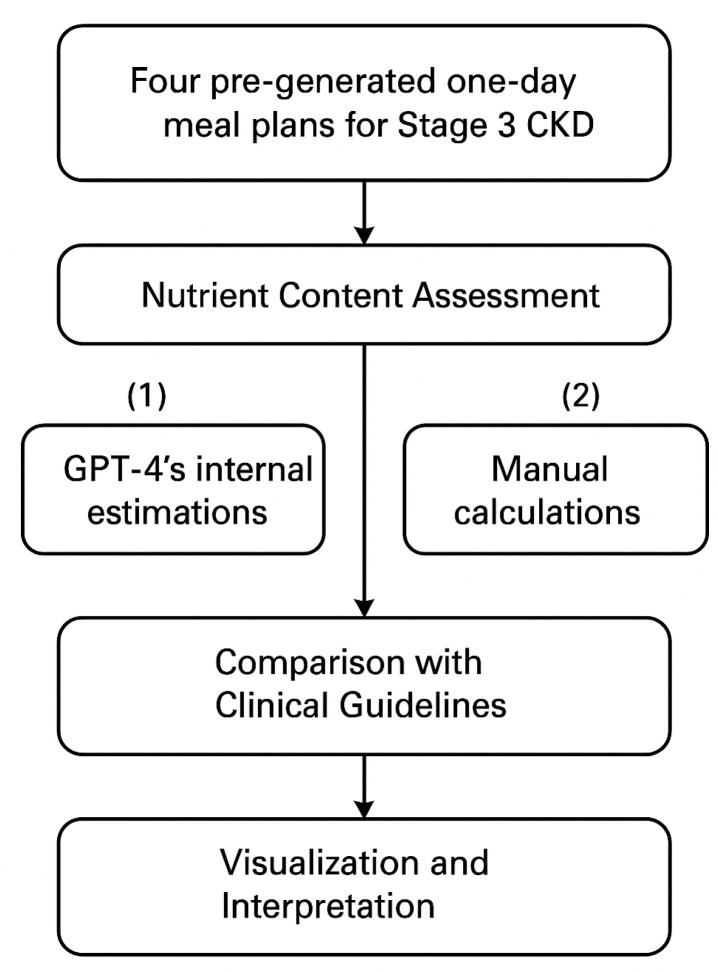
Framework of the stepwise nutritional component analysis used to evaluate AI-generated and initial diets for Stage 3 CKD.

To assess the accuracy and clinical usefulness of AI-generated dietary recommendations for CKD, a multi-step nutritional analysis was conducted (Figure 2). All three models were used to generate daily meal plans for patients with all five stages of CKD taking into account all stage-specific dietary needs. Each meal plan was appraised in relation to significant nutritional components of interest to CKD progression and complications development, including protein, sodium, potassium, phosphorus, and total energy (calories). These nutrients are classified as vital based on their role in maintaining metabolic balance and protecting against disease-caused complications such as hyperkalemia, fluid overload, and mineral bone disease.

Although similar procedures were applied to meal plans developed for CKD Stages 1, 2, 4, and 5, the findings reported here focus exclusively on Stage 3 CKD, as this stage is considered as the most common stage across general population. At stage 3, kidney damage is significant, but not yet severe, this allows testing of interventions (diet, lifestyle, medications) aimed at slowing decline of kidney functions and this stage allows safer trial of interventions compared to 4–5 stages [36]. For CKD Stages 1, 2, 4, 5 descriptive nutritent checks were performed (Table A3, Appendix B). All LLM outputs across CKD stages are publicly available at our GitHub repository (see Data availability Section). This stage was selected due to its clinical importance in early intervention and dietary modification before dialysis becomes necessary. In the case of Stage 3 CKD, four pre-formulated one-day diet plans were contrasted: the patient’s baseline diet and three AI-formulated plans created by GPT-4, Gemini, and Copilot. The whole nutritional component analysis can be summarized in three stages:

1. Nutrient Quantification

Each diet was analyzed for total energy (kcal) and key nutrients relevant to CKD management, including protein, sodium, potassium, and phosphorus. Nutrient values were obtained using two approaches:

- Manual calculation, based on USDA FoodData Central [37] and renal-specific dietary sources such as DaVita Kidney Care and the National Kidney Foundation [38,39].
- GPT-4’s internal estimations, generated for each provided diet plan. It was chosen for this role based on its demonstrated consistency in structured outputs and its established use in prior studies as an evaluation model [40].

2. Comparison with Clinical Guidelines

Nutrient values were assessed for alignment with established dietary recommendations for Stage 3 CKD, as summarized in Table A4 (Appendix B). Each nutrient was categorized as “below,” “within,” or “above” the target range to evaluate guideline adherence. Table A4 (Appendix B) summarizes the key dietary recommendations extracted from multiple clinical guidelines, serving as the benchmark for assessing the AI-generated diets. By systematically comparing the generated meal plans against these established standards, this study aimed to determine the level of adherence to medical nutrition therapy principles.

3. Visualization and Statistical Analysis

The manually calculated nutrient values and GPT-4 estimations were compared using descriptive statistics, including mean, standard deviation, mean difference, maximum absolute difference and error analysis. Bar graphs, comparative charts, and Bland-Altman plots were used to visualize variations across diets. Special attention was given to nutrients with potential clinical risk if misestimated, such as protein, phosphorus, and potassium. This multi-method analysis provided a comprehensive view of how well AI-generated recommendations align with evidence-based nutritional therapy standards and whether discrepancies exist between GPT-4 estimations and manual calculations.

In addition to statistical and nutrient analysis, a brief qualitative analysis of generated meal plans was performed to identify examples of suggested foods that may present challenges in terms of local accessibility or everyday use (Appendix B, Table A8).

#### 2.3.5. Statistical Significance

A *p*-value of <0.05 was considered statistically significant for all inferential analyses.

### 2.4. Ethical Approval

The present study utilized five mock patient profiles to generate AI-based dietary recommendations for individuals with CKD. As the study does not involve real patient data, ethical approval was not required from the Ethics Committee. The mock profiles were designed to reflect diverse clinical and dietary characteristics relevant to CKD management, ensuring a comprehensive evaluation of the AI-generated recommendations.

## 3. Results

### 3.1. Descriptive Statistics

**Figure A1:**
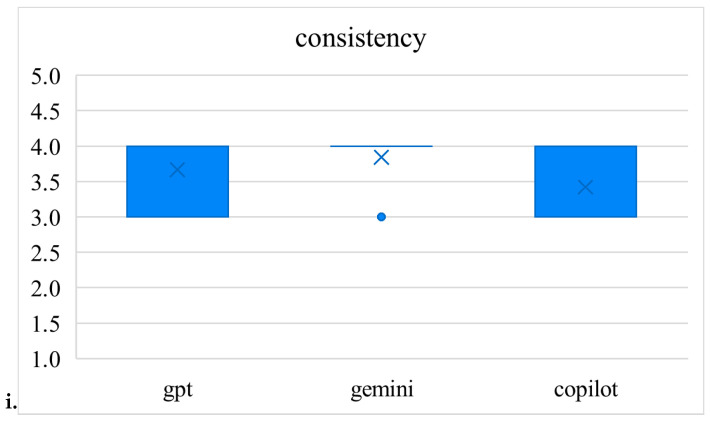

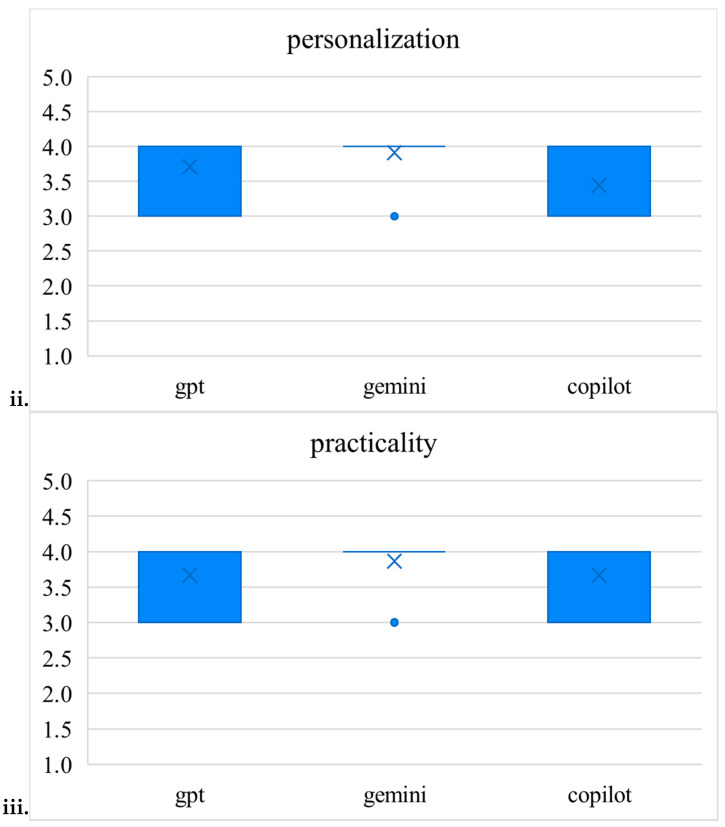
(**i**–**iii**) Box Pot Representation of AI Evaluation Scores by Model and Criteria (× = mean, line = median, • = outlier).

The results below compare dietary recommendations generated by Copilot, Gemini, and ChatGPT-4 for five CKD cases, evaluated across three criteria: consistency, practicality, and personalization. The median evaluations scores along with IQR, mean ± SD, minimum and maximum scores, number of ratings by model and criterion (*n*) are summarized in Table 1. Box plot representations of the scores by model and criteria are presented in Figure A1 (Appendix B).

As indicated in Table 1, Gemini demonstrated the highest mean scores across all three criteria, particularly in personalization, where it received an average score of 3.91 ± 0.29. ChatGPT-4 followed, performing comparably well in practicality and consistency, with mean scores of 3.67 ± 0.48 in both categories. Copilot, while achieving slightly lower scores than Gemini and GPT-4, still maintained a relatively strong performance across all three measures.

Among the three evaluation criteria, practicality was rated higher than consistency and personalization in the case of Copilot. However, for GPT-4 and Gemini, the differences across criteria were minimal, suggesting that while AI-generated recommendations were generally feasible for real-world application, there was slightly more variation in their ability to remain consistent and personalized across different CKD patient profiles.

### 3.2. Statistical Analysis

To assess differences in AI-generated nutritional recommendations, a Kruskal-Wallis test was performed to compare consistency, practicality, and personalization scores across the three AI models (Copilot, Gemini, and GPT-4). The results indicate significant differences in consistency (χ^2^ = 17.520, df = 2, *p* < 0.0002) and personalization (χ^2^ = 22.848, df = 2, *p* < 0.0001) across AI models, with Gemini consistently achieving the highest scores (Table 2). Differences in practicality were marginally significant when adjusting for ties (χ^2^ = 6.091, df = 2, *p* < 0.0476), suggesting that Gemini may also have an advantage in this aspect.

These findings indicate that Gemini has the most consistent and personalized dietary advice, with Copilot typically having the lowest scores.

To compare differences in practicability, consistency, and personalization among the AI tools (GPT-4, Gemini, and Copilot), Dunn’s post hoc test with Bonferroni adjustment was applied following Kruskal–Wallis test. Results of pairwise comparisons along with z-test values and corresponding *p*-values are shown in Table 3.

Analysis of the data uncovered substantial variations in personalization across the three models of AI. Gemini exhibited a much greater degree of personalization than Copilot (z = −4.7638, *p* < 0.0001), and GPT-4 also showed an improvement over Copilot (z = −2.7222, *p* < 0.0097). Although GPT-4 appeared to provide less personalized responses compared with Gemini, this difference did not reach statistical significance (z = −2.0416, *p* = 0.0618).

A similar trend was observed for consistency. Gemini showed significantly greater consistency than Copilot (z = −4.1684, *p* < 0.001), while GPT-4 was also significantly more consistent than Copilot (z = −2.4133, *p* < 0.0237). When comparing GPT-4 and Gemini, Gemini showed higher consistency, but this difference did not reach statistical significance (z = 1.7551, *p* < 0.1189).

Regarding practicality, Gemini again outperformed Copilot (z = −2.1373, *p* < 0.0489), while GPT-4 and Copilot did not show a significant difference (z = 0.0000, *p* > 0.5). However, GPT-4 performed slightly better than Gemini in this category (z = 2.1373, *p* < 0.0489), suggesting that GPT-4’s recommendations may be more feasible for implementation in real-world settings, despite Gemini’s overall stronger performance.

To quantify the differences between the groups, effect sizes were calculated using Cliff’s delta (δ) for all pairwise model comparisons across personalization, consistency, and practicality (Appendix B, Table A5). Most effects were small, such as GPT versus Gemini for personalization (δ = 0.20), consistency (δ = 0.18), and practicality (δ = 0.20); GPT versus Copilot for personalization (δ = −0.27), consistency (δ = −0.24), and practicality (δ = 0); and Gemini versus Copilot for practicality (δ = −0.20), indicating modest differences between models, with two medium effects observed for Gemini versus Co-pilot in personalization (δ = −0.47) and consistency (δ = −0.42), highlighting Gemini’s clear advantage over Copilot in these areas.

### 3.3. Inter-Rater Reliability

To assess the level of agreement among the three expert evaluators on the AI-generated dietary recommendations, Krippendorff’s Alpha was calculated for each evaluation criterion: personalization, consistency, and practicality. As shown in Table A6 (Appendix B), inter-rater reliability in personalization was α = 0.22, which was a measure of fair rater agreement. This is a measure of a low to moderate degree of consistency between raters’ perception of the degree of personalized dietary change from different chatbot responses. In consistency, which measured adherence to pre-established clinical nutrition standards, Krippendorff’s Alpha was 0.11, with agreement only being low. Lowest agreement was observed in the practicality dimension, with an alpha of −0.20, indicating poor or no agreement among the examiners.

### 3.4. Nutritional Component Analysis

Table 4 presents the nutrient composition of four daily diet plans: Initial, GPT-4, Gemini, and Copilot based on manual and ChatGPT-4 -generated calculations. Each plan was evaluated for energy content (kcal) and the intake of four key nutrients relevant to CKD Stage 3 management: protein, sodium, potassium, and phosphorus. Manually calculated nutrient estimates showed the following results:

Protein intake in the Initial (95.4 g), Copilot (95.9 g), and Gemini (102.1 g) plans was above the optimal 50–66 g/day for CKD Stage 3 patients. Only the GPT-4 plan was within target, at 54.0 g.

Sodium intake in all plans was well below the recommended upper limit of 2300 mg/day, ranging from 731 mg (GPT-4 plan) to 1326 mg (Copilot plan). Potassium intake was the least in the GPT-4 plan (1373 mg), with the Initial (1541 mg) and Copilot (1756 mg) plans behind, all lower than the recommended 2000–3000 mg/day. The only plan that met the potassium guideline was the Gemini plan, with a combined total of 2604 mg based on manual calculations.

**Figure A2:**
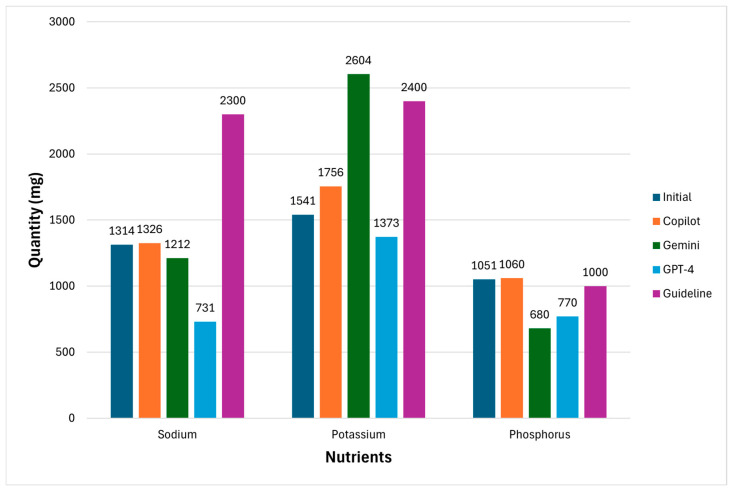
Comparison of Nutrient Estimates: Manual vs. Guidelines for Four Daily Diet Plans.

**Figure A3:**
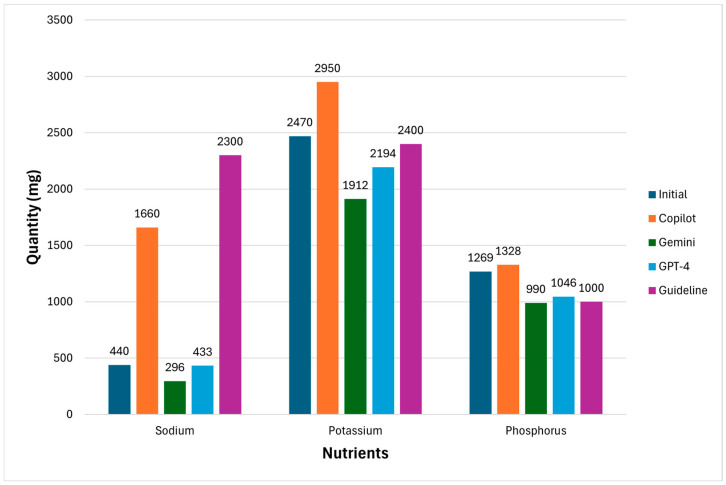
Comparison of nutrient estimates: ChatGPT4 generated vs. Guideline estimates for four meal plans.

Phosphorus intake was highest in the Copilot (1060.0 mg) and Initial (1051.0 mg) plans, both exceeding the recommended upper limit of 1000 mg/day. The GPT-4 plan (770.0 mg) and Gemini plan (680.5 mg) remained within acceptable phosphorus levels. These differences are visually represented in Figure A2 and Figure A3 (Appendix B), which compares nutrient estimates across the four plans using manually calculated data versus GPT-4 outputs.

GPT-4’s automated nutrient analysis for the same four diet plans reveled the following:

Protein content was classified by GPT-4 as above target in all plans except the Gemini plan (58 g), which was deemed within range. Sodium intake was well below the 2300 mg/day threshold in all plans, with especially low values in the Initial (440 mg), Gemini (296 mg), and GPT-4 (433 mg) plans.

Potassium levels were generally within the target range of 2000–3000 mg/day, except for the Gemini plan (1912 mg), which GPT-4 flagged as slightly low. At the same time, the Copilot plan achieved the higher limit (2950 mg) but did not exceed it.

Phosphorus intake was higher than the recommendation range (800–1000 mg/day) on the Initial (1269 mg), Copilot (1328 mg), and GPT-4 (1046 mg) plans. All the remaining Gemini plan remained within the advisory phosphorus content (990 mg).

These automated tests show how GPT-4 can classify nutrient intake against clinical cut-points and present an instant guideline-directed interpretation of nutritional sufficiency for CKD care.

To assess the accuracy and consistency of GPT-4’s estimates of nutrients, summary statistics were contrasted with manually calculated values (Table 5 and Table 6). Table 5 provides plan-level absolute and percentage errors, demonstrating that the largest discrepancies occurred for potassium (1194 mg, 68% in the Copilot plan) and sodium (916 mg, −75.58% in the Gemini plan). Errors for protein ranged from −6.71% to +53.7%, and for phosphorus from 20.74% to 45.59%. Notably, some plans exceeded guideline thresholds for phosphorus (Copilot, GPT-4) or approached the upper limit for potassium (Copilot), highlighting potential safety risks if applied without professional oversight. Additional details on error distribution, including mean absolute percentage error with 95% confidence intervals, are provided in Appendix B, Table A7.

When averaged across the four plans, Table 6 summarizes mean differences in nutrient estimates. GPT-4 low-estimates protein by −7.60 g and sodium by −438.50 mg, respectively, using average mean differences. GPT-4 over-estimated potassium and phosphorus content by +563.00 mg and +267.88 mg, respectively.

Standard deviations indicated great variation in estimates of sodium by GPT-4 (SD = 638.62 mg) compared with the manual method (SD = 281.19 mg), which suggests uneven sodium prediction among plans. The greatest absolute differences were extremely large for potassium (1194 mg) and sodium (916 mg), which suggests potential clinically meaningful variability.

**Figure A4:**
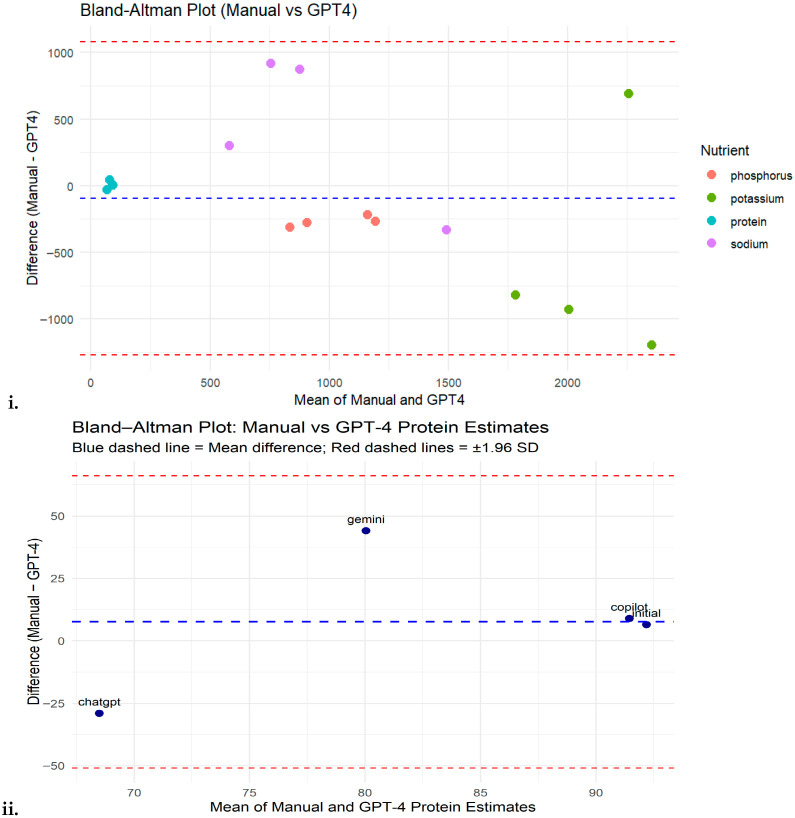
Bland–Altman plots comparing manual and GPT-4 nutrient estimates: (**i**) all nutrients (protein, sodium, potassium, phosphorus) and (**ii**) protein only.

These results emphasize that while GPT-4 provides overall trends consistent with human assessment, quantitative differences among specific nutrient estimations, particularly sodium and potassium, require careful evaluation before use of AI-developed meal plans in clinical application. Additional visual agreement between manual and GPT-4 calculated nutrient estimates is shown in a Bland-Altman plot (Appendix B, Figure A4i,ii). It showed close agreement between GPT-4 and manual calculations for protein, while plots for all nutrients revealed wider limits of agreement for sodium and potassium, indicating greater variability and systematic underestimation, with moderate overestimation for phosphorus.

### 3.5. Qualitative Analysis of AI-Generated Meal Plans

Brief qualitative analysis, conducted under practicality and availability criteria, revealed several food items within each generated meal plan were less feasible for regular dietary use. For example, brown rice was recommended by all three AI models across different stages, despite being rarely purchased in daily practice; quinoa and tofu appeared in outputs from Copilot and Gemini, although they are not part of routine local diets; and almond milk and low-phosphorus milk alternatives were suggested by Gemini and ChatGPT-4, yet these products are generally expensive and not widely available. In addition, hummus was proposed by Copilot and ChatGPT-4, although it is not commonly consumed. These examples (summarized in Table A8, Appendix B) illustrate how nutritionally adequate outputs may face practical barriers when assessed in terms of availability and cultural relevance.

## 4. Discussion

The findings of this study emphasize both the promise and the ongoing limitations of LLM chatbots in providing dietary recommendations for CKD. Although certain AI models showed an ability to propose balanced meal plans roughly consistent with established guidelines, there remain critical moments when the chatbot advice faltered, particularly regarding sodium, potassium, and phosphorus amounts, nutrients that can pose acute risks if miscalculated for CKD patients. In this study, ChatGPT-4 was used to calculate nutrient content of AI-generated meal plans produced by different models. In contrast to manual calculations, ChatGPT-4 demonstrated significant overestimation of potassium levels and underestimation of sodium levels of meal plans by 60% and 40% in certain instances. Given these inaccuracies in nutrient values, ChatGPT-4’s nutrient estimations should be considered as preliminary assessments rather than definitive calculations, as these inaccuracies may have clinically significant implications in dietary planning for individuals with CKD. In particular, they may risk inadequate sodium control or hyperkalemia, which is associated with the development of cardiac arrythmias and cardiac arrest [45]. Recognizing these risks underscores the importance of careful dietary management and close monitoring in CKD populations. Therefore, comparison with a deterministic, validated nutrient analysis tools remain essential prior to clinical implementation. These observations closely align with multiple recent investigations. Kim et al. concluded that AI-generated weight-management plans can, at times, be indistinguishable from those created by dietitians, yet also found that the absence of precise details, such as exact portion sizes or affordability considerations, can impair real-world applicability [33]. Similarly, Ponzo et al. reported that even when ChatGPT provided solid nutritional suggestions for various NCDs, its reliability waned in complicated cases, consistent with the challenges we observed when prompting the model to handle overlapping restrictions such as fluid, sodium, and potassium constraints [23].

In yet another study focusing on Central Asian contexts specifically, Adilmetova et al. noted that ChatGPT can work significantly differently from language to language, with significantly lower quality in Kazakh compared to English and Russian. They attributed these variations as a consequence of the lack of adequate localized training data for under-resourced languages and therefore underscore the greater imperative toward culturally and linguistically aligned AI solutions [29]. This conclusion concurs with our own experiences of chatbot restrictions on area-based diets and ingredients, to the extent of corroborating the premise that certain nutritional advice might be impractical without detailed local context.

Where other systems partially bridge such gaps, quiet errors still occur. Ponzo et al. have documented fluctuation in nutritional advice between back-to-back chat sessions, illustrating how the same question over two successive days or the same prompts can yield differing responses [46]. Similar results have been observed in other studies, where the alterations in prompt formulations resulted in the varied accuracy and consistency levels [47,48]. For instance, Wang et al. observed variable agreement with clinical guidelines depending on prompt style, and Azimi et al. found that prompting strategies such as Chain-of-Thought or Retrieval-Augmented Prompts significantly shifted performance levels across nutrition exam questions [47,48]. This highlights the issue of prompt sensitivity, which may also influence findings in the present study given that a single standardized prompt was employed [49]. In addition to prompt sensitivity issues, other studies report instances of clinically important inaccuracies. Wang et al. also documented meal plans that were given to patients on dialysis underpredicted daily loads of nutrients, potentially leading to negative long-term outcomes [18]. Even so, chatbots are readably written: Pugliese et al. were able to prove that patients who had nonalcoholic fatty liver disease (NAFLD) evaluated AI response as readable and enjoyable, which was also identified as a property in our work, where users labeled the AI-produced text readability [50]. However, as noted by Kim et al. and Garcia, friendly and plain language is no promise of complete adherence to clinical best practices, especially to those who have high needs [16,33].

Other researchers have proposed hybrid models that can optimize overall performance. Papastratis et al. combined deep generative models with a rules-based model of nutrition and demonstrated that strong guideline constraints can reduce the frequency of risky or incompatible meal suggestions [22]. Ponzo et al. and Niszczota & Rybicka have likewise emphasized the significance of “prompt engineering,” where specifying nutrient cutoffs or target macronutrient percentages in the question can noticeably improve alignment with clinical goals [23,51]. While helpful, these extra steps demand that users or clinicians already possess a certain level of knowledge to guide the AI effectively, mirroring the practical dilemmas that Johnson et al. highlight about users potentially misunderstanding or misapplying AI advice [52]. Similar concerns are raised in the comparative analyses by Lo et al. and the Bragazzi et al., both of which found that AI-driven responses can appear quite authoritative yet contain subtle factual slips [24,53]. Hieronimus et al. demonstrated that ChatGPT and Bard (now called Gemini) can produce nutritionally adequate meals for certain dietary patterns, but also noted frequent shortfalls in meeting micronutrient recommendations, a risk that can be consequential for patients requiring precise dietary management, such as those living with CKD [54].

One of the most persistent issues is the failure of the chatbots to properly localize to socioeconomic or cultural environments, reducing adherence to suggested plans. CKD patients in real-world settings might not be able to obtain fresh vegetables or specialty low-sodium foods. Studies like Kim et al. show that exclusion of cost consideration, food availability, or traditional habits reduces real-world compliance [33]. Use of real-time data merging is a major concern as well. Qarajeh et al. suggested that chatbots linked to dynamic clinical laboratory results may be able to better adjust fluid or protein limitations in near-real-time, but it is still challenging to achieve that degree of synergy between platforms [25]. That gap may prove crucial, because CKD dietary management often requires continuous recalibration based on changes in estimated glomerular filtration rate (eGFR) or potassium levels.

Although safety and liability concerns abound, the potential of AI for patient education and streamlined diet counseling continues to attract attention. Garcia underlines that chatbots can enhance nutrition literacy among general consumers, especially where access to a dietitian is limited [16]. Yet Kim et al. and Ponzo et al. warn that chatbots seldom accept legal responsibility for harmful suggestions, leaving the burden on patients or clinicians to detect subtle miscalculations in nutrient content [33,46]. The same tension emerges in both research and clinical practice: on one hand, the ability to scale free, interactive diet advice may help reduce disparities, but on the other, the risk of following errors or oversights could have dangerous health effects.

Sustained efforts at integrating strong rule sets and knowledge bases specific to domains into these AI systems would be helpful. Papastratis et al. illustrated that giving ChatGPT an “almost infinite” list of potential meals but then running them through a strong algorithmic infrastructure yielded more accurate daily meal plans for obesity, diabetes, and cardiovascular disease [17]. For CKD, the same approach might include each phase’s individual protein, phosphorus, and potassium limits, rejecting or rewording meal suggestions that exceed clinically recommended limits.

Côté & Lamarche argue that broad improvements in AI’s transparency, where the model shows how it arrived at a particular recommendation, could further empower dietitians to refine the plan [20]. However, the technique known as “explainability” or “interpretability” in AI remains relatively immature in these generative chatbot frameworks, an ongoing barrier to widespread acceptance.

In short, the combined evidence from this study and related work underscores that LLM-driven dietary guidance can be a helpful, readily accessible supplement for CKD and other chronic conditions but is not yet equipped to displace professional clinicians. Numerous studies indicate a future where AI largely offloads the task of routine patient education and meal planning, but with clinicians still holding ultimate responsibility for high-stakes recommendations [23,33,46,54]. Other studies also highlight its potential in fields such as cardiology, highlighting its potential in ECG interpretation and arrythmia detection, while emphasizing that human clinicians are essential for oversight, decision-making, and monitoring AI-supported interventions [55]. The importance of human oversight was also emphasized in a recent study by Sblendorio et al., which showed that integrating expert review with automated evaluation across multiple domains—such as safety, accuracy, ethics, and consistency—enhances the reliability and clinical feasibility of LLM [56]. A model like this could couple the generality of generative AI with judiciously inserted constraints on the amount of nutrients, along with timely human oversight. Ultimately, prospective, large-scale trials that measure patient outcomes are needed to confirm the actual benefits and hazards of AI-driven dietary advice for CKD. Until then, the prudent course is to treat chatbots as valuable but inherently fallible assistants: a means of supplementing, not supplanting, the expertise of registered dietitians and nephrologists in safeguarding the delicate health of individuals with kidney disease.

A primary strength of this study is the direct comparison of three prominent AI chatbots (GPT-4, Gemini, Copilot) to determine their capacity for producing culturally relevant and clinically aligned dietary recommendations for a complex condition such as CKD. This approach draws on methods from comparable research [23,29,33] but places distinct emphasis on Central Asian dietary habits and local feasibility factors, aspects frequently overlooked in studies of AI nutrition tools [17,51]. In addition, the standardized set of profiles representing CKD stages 1 through 5 is intended to reflect the variety of real-world conditions, mirroring the complexity seen in clinical settings.

Several limitations must be noted. The study relied on mock patient profiles rather than actual clinical or laboratory data, which may limit the AI’s capacity to adjust recommendations in response to fluctuations in serum potassium, eGFR, or other key lab indicators [18,25]. While clinically designed and validated for accuracy and dietary relevance, these simulated profiles cannot fully reflect the variability and complexity of real CKD patients, which may limit generalizability of findings. The use of a single standardized prompt is another limitation, as prior studies have shown that LLM outputs can vary considerably with different prompt formulations [47,48,49]. Future work should therefore include sensitivity analyses to systematically assess prompt variability and better quantify prompt sensitivity. In addition, inter-rater disagreement emerged, in particular for practicality, meaning that different experts interpreted the same AI suggestions in quite different ways. Similar issues have been documented by Ponzo et al. [46] and may point to the need for standardized evaluation rubrics or more explicit rating instructions regarding the real-world feasibility of meal plans [33]. A five-point Likert scale was employed for aggregated scoring, but this may not fully capture subtle differences in cultural contexts or personal preferences [54]. At the same time, as this study relied on expert ratings, it remains partly subjective. Recent studies have shown that integrating computational text analyses—such as semantic or lexicometric evaluation—can enhance objectivity and reproducibility in assessing AI-generated health texts [57,58]. Incorporating such approaches in future research could strengthen the robustness of evaluation frameworks. The chatbots used are also not trained on detailed local ingredient databases, limiting their effectiveness for region-specific diets [29,33]. Moreover, nutritional analysis in this study mainly focused on Stage 3 CKD due to its prevalence and clinical relevance, while only descriptive nutrient checks were provided for other stages. This might limit the generalizability of findings, and future studies should incorporate more systematic analyses across all CKD stages to fully establish clinical utility. Finally, liability remains with the user or medical professional, as chatbots themselves disclaim responsibility for any inaccuracies or omissions, which reflects a broader pattern in AI-assisted healthcare [16,52].

## 5. Conclusions

The study’s findings suggest that AI chatbots are capable of generating meal plans that sometimes comply with established CKD dietary guidelines, indicating a possible role in providing quick, accessible nutrition information. However, inconsistencies commonly arose in critical nutrient suggestions, and both cultural adaptation and practical implementation proved to be vulnerable aspects, as suggested by the negative reliability metrics for practicality. Similar observations have been made in research emphasizing the need for oversight when AI chatbots are used in high-stakes clinical scenarios such as CKD.

A strengthened design may be achieved by incorporating region-specific data, real-time lab integration, and robust rule-based frameworks, to improve both reliability and cultural relevance. Even so, guidance by registered dietitians and nephrologists remains critical, given the fine margins of error in CKD nutritional management. While AI-driven methods hold promise for enhancing patient education and reducing clinical workload, current findings and related literature underscore that professional judgment remains essential in ensuring each patient’s specific needs are appropriately addressed.

## Figures and Tables

**Table 1:** Assessment of AI Models for Nutritional Advice: Summary of Evaluation Results (135 ratings per criterion, across CKD stages 1–5).

AI Model	Criterion	Median (IQR)	Mean ± SD	Min	Max	*n*
ChatGPT-4	Consistency	4 (1)	3.67 ± 0.48	3.00	4.00	45
	Practicality	4 (1)	3.67 ± 0.48	3.00	4.00	45
	Personalization	4 (0)	3.71 ± 0.46	3.00	4.00	45
Gemini	Consistency	4 (0)	3.84 ± 0.37	3.00	4.00	45
	Practicality	4 (0)	3.87 ± 0.34	3.00	4.00	45
	Personalization	4 (0)	3.91 ± 0.29	3.00	4.00	45
Copilot	Consistency	3 (1)	3.42 ± 0.50	3.00	4.00	45
	Practicality	4 (1)	3.67 ± 0.48	3.00	4.00	45
	Personalization	3 (0)	3.44 ± 0.50	3.00	4.00	45

**Table 2:** Kruskal–Wallis Analysis of Consistency, Practicality, and Personalization Scores Across Three AI Models.

Criterion	AI Model	Rank Sum	χ^2^ (df = 2)	*p*-Value
Consistency	ChatGPT-4	3127.5	17.52	0.0002 *
	Gemini	3667.5		
	Copilot	2385		
Practicality	ChatGPT-4	2857.5	6.091	0.0476 *
	Gemini	3465		
	Copilot	2857.5		
Personalization	ChatGPT-4	3127.5	22.848	0.0001 *
	Gemini	3735		
	Copilot	2317.5		

* Statistically significant result at 0.05 level.

**Table 3:** Dunn’s Post-Hoc Pairwise Comparisons for Personalization, Consistency, and Practicality Among AI Models.

Comparison	Personalization		Consistency		Practicality	
	z-Test	*p*-Value	z-Test	*p*-Value	z-Test	*p*-Value
GPT-4–Gemini	2.0416	0.0618	1.7551	0.1189	2.1373	0.0489 *
GPT-4–Copilot	−2.7222	0.0097 *	−2.4133	0.0237 *	0	1
Gemini–Copilot	−4.7638	0.0001 *	−4.1684	0.0001 *	−2.1373	0.0489 *

* Statistically significant result at 0.05 level.

**Table 4:** Manually Calculated vs. ChatGPT-4—Generated Nutrient Composition of Daily Diet Plans with CKD Stage 3 Guideline Classification.

Diet	Protein (g)		Sodium (mg)		Potassium (mg)		Phosphorus (mg)	
	Manual	ChatGPT-4	Manual	ChatGPT-4	Manual	ChatGPT-4	Manual	ChatGPT-4
Initial	95.4 ↑	89 ↑	1314	440	1541	2470 ↑	1051 ↑	1269 ↑
ChatGPT-4	54	83 ↑	731	433	1373	2194	770	1046 ↑
Gemini	102.1 ↑	58	1212	296	2604	1912	680	990
Copilot	95.9 ↑	87 ↑	1326	1660	1756	2950 ↑	1060 ↑	1328 ↑
Guideline [2,41,42,43,44]	58		2300 (limit)		2400 (limit)		1000 (limit)	

Note: ↑ indicates above the target, blank indicates within the recommended range.

**Table 5:** Comparison of GPT-4 and Manual Nutrient Estimates with Absolute and Percentage Errors.

	Protein		Sodium		Potassium		Phosphorus	
Diet	Absolute Error	% Error	Absolute Error	% Error	Absolute Error	% Error	Absolute Error	% Error
Initial	6.40	−6,71%	874	−66.51%	929	60.29%	218	20.74%
ChatGPT-4	29	53.70%	298	−40.77%	821	59.80%	276	35.84%
Gemini	44.10	−43.19%	916	−75.58%	692	−26.57%	310	45.59%
Copilot	8.90	−9.28%	334	25.19%	1194	68%	268	25.28%

**Table 6:** Statistical Comparison of GPT-4 and Manually Calculated Nutrient Values Across Four Diet Plans.

Nutrient	ChatGPT-4		Manual		Mean Difference (GPT4−Manual)	Max Abs Difference
	Mean	SD	Mean	SD		
Protein (g)	79.25	14.38	86.85	22.11	−7.60	44.1
Sodium (mg)	707.25	638.62	1145.75	281.19	−438.50	916
Potassium (mg)	2381.50	442.2	1818.50	546.62	563	1194.00
Phosphorus (mg)	1158.25	165.32	890.38	194.17	267.88	309.5

## Data Availability

The mock patient cases, evaluation criteria, completed evaluations, and prompts used in this study will be made publicly and freely available without restriction after the publication of the manuscript at https://huggingface.co/datasets/issai/llm_for_ckd.

## Abbreviations

The following abbreviations are used in this manuscript:

AI	Artificial Intelligence
BMI	Body Mass Index
CKD	Chronic Kidney Disease
EJCN	European Journal of Clinical Nutrition
GPT	Generative Pre-trained Transformer
GPT-4	Generative Pre-trained Transformer, version 4
KDIGO	Kidney Disease: Improving Global Outcomes
KDOQI	Kidney Disease Outcomes Quality Initiative
LLM	Large Language Model
NAFLD	Non-Alcoholic Fatty Liver Disease
NCP	Nutrition Care Process
SD	Standard Deviation
USDA	United States Department of Agriculture
Stata MP/18	Stata Multiprocessor Edition, Version 18

## Appendix A. Standardized Mock Patient Profiles CKD Stages 1 to 5

**Patient with Stage 1 Chronic Kidney Disease (CKD) and Type 2 Diabetes Mellitus (DM)**
  **Name:** Aizhan
  **Gender:** Female
  **Age:** 55
  **Nationality:** Kazakhstani
  **Location:** Almaty, Kazakhstan
  **Family Information:**
  **Marital Status:** Married
  **Family Members:** Husband (Nursultan, 58), Son (Arman, 30)
  **Occupation: Accountant**
  **Cultural Background:** Kazakh, fluent in Kazakh and Russian. Follows a traditional Kazakh diet with heavy emphasis on meat (especially lamb and beef), dairy products (like kefir and cottage cheese), and bread. Vegetables are included but less frequently, especially in winter months when fresh produce is harder to obtain.
  **Medical Information:** Diagnosis: Type 2 Diabetes. Chronic kidney disease, stage 1. Hypertension.
  **Date of Diagnosis:** January 2024
  Hypertension (diagnosed 8 years ago)
  Type 2 Diabetes Mellitus (diagnosed 6 years ago)
  **Medical History:**
  Diagnosed approximately 6 years ago after a routine blood test revealed elevated blood sugar levels. Aizhan’s kidney function is monitored through regular blood and urine tests to check for any changes in eGFR or albumin levels, which have remained stable so far. Aizhan’s father had cardiovascular disease and passed away at age 65 from a heart attack, while her mother has been living with type 2 diabetes for over 20 years and has mild kidney dysfunction.
  **Current Medications:**
  Enalapril 10 mg once daily
  Metformin 500 mg twice daily (with meals)
  Aspirin (Low-dose) (for cardiovascular protection) 75 mg once daily
  Vitamin D (Cholecalciferol) 1000 IU once daily
  Atorvastatin 10 mg once daily
  **Diet History:**
  **Breakfast:** Green tea, kefir and whole-grain bread with a small amount of butter or cheese.
  Occasionally, has fried eggs with salad (cucumbers and tomatoes) when she feels hungry.
  **Lunch:** Plov (Kazakh rice dish with lamb, onions, and carrots). A side of pickled vegetables (cabbage or cucumbers). Drinks green tea or water with lunch.
  **Dinner:** Roast chicken with baked vegetables (like carrots, pumpkins, and bell peppers). Often has fresh bread on the side but tries to limit it. A small portion of fruit, typically an apple or orange.
  **Environmental, Behavioral, and Social Factors:** Resides in Almaty, Kazakhstan, a city with easy access to medical care, though it can sometimes be difficult to navigate the public healthcare system. Walks a few times a week, but due to a sedentary job, doesn’t engage in regular physical exercise. Non-smoker, drinks alcohol socially on weekends (typically vodka or wine). Strong family ties; her son Arman and husband Nursultan are supportive, but they have limited knowledge of managing chronic diseases.
  **Assessment:**
  **Anthropometry, Body Composition, and Functional:**
  Weight: 185 lbs (84 kg)
  Height: 5′5” (165 cm)
  BMI: 31.5 (Obese)
  **Biochemical and Hematological Markers:**
  Serum Creatinine (SCr) 0.77 mg/dL.
  GFR (Glomerular Filtration Rate): 91 mL/min/1.73 m^2^ (CKD-EPI)
  Hemoglobin A1c: 7.5%
  Sodium: 140 mEq/L
  Potassium: 4.4 mEq/L
  Albumin/Creatinine Ratio: 300 mg/g
  Blood Pressure: 135/85 mmHg
  Total Cholesterol: 220 mg/dL
  LDL Cholesterol: 130 mg/dL
  HDL Cholesterol: 50 mg/dL
  Triglycerides: 160 mg/dL
  Non-HDL Cholesterol: 170 mg/dL
  Urine output 1.2 to 2 L per day.
  **Additional Information:** Aizhan’s CKD is currently classified as stage 1 (89 mL/min/1.73 m^2^), with mild proteinuria and stable kidney function. However, her history of diabetes and hypertension put her at an increased risk for progression.
  **Patient with Stage 2 Chronic Kidney Disease**
  **Name:** Aida
  **Gender:** Female
  **Age:** 53
  **Nationality:** Kazakhstani
  **Location:** Almaty, Kazakhstan
  **Family Information:**
  **Marital Status:** Married
  **Family Members:** Husband, no children
  **Occupation:** Cashier
  **Cultural Background:** Aida is a native Kazakh and has lived her entire life in Kazakhstan. She follows Islamic practices and has a deep respect for her cultural traditions, which include strong ties to community and family. Her diet is influenced by Kazakh cuisine, known for hearty dishes that feature meat, bread, and dairy products.
  **Medical Information:** Diagnosis: Chronic kidney disease, stage 2. Concomitant: Hypertension secondary to other renal disorders.
  **Date of Diagnosis:** 2 years ago
  **Medical History:** Aida’s CKD was discovered during a routine health check-up, which showed an eGFR (estimated Glomerular Filtration Rate) of 60 mL/min/1.73 m^2^. Her initial symptoms were subtle, including mild fatigue and slight swelling in her ankles, which she attributed to her busy teaching schedule and long working hours.
  **Current Medications:** Amlodipine 5 mg daily, atorvastatin 10 mg daily, vitamin D 1000 IU daily. Lisinopril 20 mg daily.
  **Diet History:**
  **Breakfast:** A cup of unsweetened green tea for antioxidants and low-fat greek yogurt.
  **Lunch:** Tuna sandwich on whole-wheat bread with a side of vegetable sticks
  **Dinner:** Pasta with marinara sauce and a side of steamed vegetables
  **Environmental, Behavioral, and Social Factors:** Cultural eating patterns, such as traditional Kazakh dishes rich in meats and dairy, can impact Aida’s CKD management. Adapting her diet to include more plant-based foods, whole grains, and lean proteins while limiting high-potassium and high-phosphorus foods is key.
  **Assessment:**
  **Anthropometry, Body Composition, and Functional:**
  Weight: Current—65 kg
  Height: 1.67 m
  BMI: 23.3 kg/m^2^
  **Biochemical and Hematological Markers:** eGFR: 67 mL/min/1.73 m^2^ (Stage 2 CKD), serum creatinine: 1.0 mg/dL, slightly elevated serum creatinine levels and increased proteinuria. Cholesterol: Total 210 mg/dL, LDL 120 mg/dL (elevated).
  Blood Pressure: 130/85 mmHg
  **Additional Information:** Aida’s CKD Stage 2 is stable, with her main focus being on preventing progression to more advanced stages. She maintains a regular follow-up with her nephrologist and primary care physician to monitor her kidney function and adjust her treatment as needed. She has been advised to limit her protein intake, avoid high-sodium and processed foods, and manage her fluid intake to prevent further kidney strain.
  **Patient with Stage 3 Chronic Kidney Disease**
  **Name:** Dariya
  **Gender:** Female
  **Age:** 42
  **Nationality:** Kazakhstani
  **Location:** Kostanay, Kostanay region, Kazakhstan
  **Family Information:**
  **Marital Status:** Married
  **Family Members:** Husband, mother of two children (ages 25 and 19)
  **Occupation:** High School Teacher
  **Cultural Background:** Dariya is Christian, specifically from the Russian Orthodox tradition, her religious beliefs and practices can play a significant role in her lifestyle and health choices.
  **Medical Information:** Diagnosis: Chronic kidney disease, stage 3a. Concomitant: Hypertension secondary to other renal disorders. Complication: Anemia of chronic disease. Obesity.
  **Date of Diagnosis:** 2 years ago
  **Medical History:** Diagnosed approximately 2 years ago during a routine checkup. Her kidney function is monitored through regular blood and urine tests. Dariya’s father had cardiovascular disease, while her mother dealt with type 2 diabetes.
  **Current Medications:** Lisinopril 10–20 mg once daily. Folic Acid 400 mcg daily. Atorvastatin 20 mg once daily. Iron Supplements, Calcium Carbonate or Calcium Acetate, Vitamin D
  **Diet History:**
  **Breakfast:** Scrambled Eggs with Spinach and a Side of Whole Wheat Toast
  **Lunch:** Lettuce Wraps with grilled chicken or turkey slices, shredded carrots, and quinoa salad with cucumbers.
  **Dinner:** Vegetable Stir-Fry with tofu, snow peas, carrots, and a small serving of brown rice. Drinks like ginger tea or chamomile tea can be soothing and aid digestion.
  Environmental, Behavioral, and Social Factors: Dariya’s husband, Mikhail, works as a civil engineer in Kostanay and plays an essential role in supporting her health journey. He helps with meal preparations and encourages her to stick to her CKD management plan. Her children, Dmitry (25) and Elena (19), also assist with daily tasks and make sure she maintains a healthy lifestyle. They share in traditional meals and participate in outdoor activities that benefit her physical well-being.
  **Assessment:**
  **Anthropometry, Body Composition, and Functional:**
  Weight: Current—83 kg
  Height: 1.60 m
  BMI: 32.4 kg/m^2^
  **Biochemical and Hematological Markers:** eGFR: 48 mL/min/1.73 m^2^. (Stage 3a CKD), serum creatinine: 1.4 mg/dL, BUN: 20–30 mg/d, proteinuria, mild anemia, phosphate (slightly elevated), secondary hyperparathyroidism
  Blood Pressure: 125/80 mmHg
  **Additional Information:** Dariya was diagnosed with CKD stage 3a 2 years ago after a routine check-up revealed reduced kidney function. Her estimated glomerular filtration rate (eGFR) was between 45–59 mL/min/1.73 m^2^, indicating moderate kidney impairment. The diagnosis was a turning point, as it was the first time she recognized that she needed to make significant changes to her lifestyle to manage her health. Dariya prioritizes physical activity to maintain a healthy weight and manage her blood pressure. She enjoys morning walks around parks, as well as light exercises at home.
  **Patient with Stage 4 Chronic Kidney Disease**
  **Name:** Nurlan
  **Gender:** Male
  **Age:** 47
  **Nationality:** Kazakhstani
  **Location:** Shymkent, Kazakhstan
  **Family Information:**
  **Marital Status:** Married
  **Family Members:** Wife, a teenage daughter.
  **Occupation:** Disabled person of the 2nd group
  **Cultural Background:** Family gatherings, celebrations, and religious observances are central to Nurlan’s life. He values traditional Kazakh dishes and often participates in events that involve sharing meals with relatives and friends. However, this love for traditional food presents a challenge for managing his CKD.
  **Medical Information:** Diagnosis: Bilateral hydronephrosis. Complicated pyelonephritis associated with chronic renal stones disease. Ureteral stent. Chronic kidney disease, stage 4. Ormond’s disease. Concomitant: Type 2 Diabetes. Hypertension secondary to other renal disorders. Complication: Anemia of other chronic diseases.
  **Date of Diagnosis:** Diagnosed last year.
  **Medical History:** Nurlan has a history of UTI, occasional episodes, likely due to reduced kidney function and diabetes, with antibiotic treatment as needed. He was hospitalized in November last year with postrenal anuria, obstructive syndrome and acute pyelonephritis, a stent was installed in the right kidney for bilateral hydronephrosis.
  **Current Medications:** Fosinopril (20 mg daily), Metformin (500 mg twice daily), Simvastatin (10 mg nightly), Iron, Calcium and vitamin D supplements.
  **Diet History:**
  **Breakfast:** Cooked oats with a bit of unsalted chicken broth and fresh cucumber and tomato salad. Green tea with no added sugar.
  **Lunch:** Kazakh-Style Grilled Chicken Skewers served with a small side of steamed white rice.
  **Afternoon Snack:** Low-Sodium Hummus (homemade or store-bought) with sliced bell peppers and carrot sticks for dipping.
  **Dinner:** Steamed Broccoli or Cauliflower and fresh herb salad.
  **Environmental, Behavioral, and Social Factors:** Nurlan’s family is an essential part of his life, providing emotional support and helping him manage his health.
  **Assessment:**
  **Anthropometry, Body Composition, and Functional:**
  Weight: Current—90 kg
  Height: 1.81 m
  BMI: 27.5 kg/m^2^
  **Biochemical and Hematological Markers:** creatinine 250.00 mmol/L (increased), elevated eGFR: 27 mL/min/1.73 m^2^, hemoglobin level 97 g/L (anemia), hyperlipidemia, hypoglycemia (reduced to normal range after treatment)
  Blood Pressure: 135/85 mmHg
  **Additional Information:** According to health assessment that included a comprehensive blood panel and urine test: significantly elevated levels of creatinine and a decrease in his glomerular filtration rate (GFR) to around 30%, indicating that his kidney function had declined to stage 4. The urine test revealed that Nurlan had proteinuria (protein in the urine), a common sign of kidney damage. The nephrologist confirmed the diagnosis of CKD stage 4 after evaluating his medical history, blood tests, and imaging studies. The findings indicated that Nurlan’s kidney function was at 25–30% of normal, with damage likely exacerbated by his preexisting hypertension and type 2 diabetes.
  **Patient with Stage 5 Chronic Kidney Disease: End-stage kidney disease (EKSD) caused by glomerular disease. Concomitant: Hypertension secondary to other renal disorders. Complication: Anemia in other chronic diseases.**
  **Name:** Ayan
  **Gender:** Male
  **Age:** 35
  **Nationality:** Kazakhstani
  **Location:** Astana, Kazakhstan
  **Family Information:**
  **Marital Status:** Single
  **Family Members:** -
  **Occupation:** Disabled person of the 1st group
  **Cultural Background:** Ayan finds comfort in prayer and the teachings of Islam. Reciting the Qur’an and participating in community prayers help him maintain a sense of peace and resilience in the face of his illness. Does not consume pork in his diet
  **Medical Information:**
  **Diagnosis:** Chronic kidney disease, STAGE 5. Terminal chronic renal failure in the outcome of glomerular kidney disease. Concomitant: Hypertension secondary to other renal disorders. Complication: Anemia in other chronic diseases.
  **Date of Diagnosis:** 3 years ago
  **Medical History:** Ayan has a history of chronic glomerulonephritis.
  **Current Medications:** Hemodialysis sessions 3 times a week for 4 h. Fosinopril 40 mg daily. Amlodipine 5 mg daily. Vitamin D-3 5000 IU. Epoetin beta 2000 IU/0.3 mL p/k p/d + Iron (III) hydroxide sucrose complex 5 mL I/V slowly. Iron, Calcium supplements
  **Diet History:**
  **Breakfast:** Oatmeal made with water, green tea (low in caffeine). Egg whites (2–3) scrambled with a few fresh herbs (parsley, dill) for flavor.
  **Lunch:** Grilled chicken breast (small portion, about 3–4 oz), seasoned with lemon juice, black pepper, and herbs. Steamed or boiled white rice with a side of steamed zucchini. A small serving of low-sodium vegetable soup made with carrots, cabbage, and a bit of dill for flavor.
  **Afternoon Snack:** Low-sodium cottage cheese (1/2 cup) with a handful of blueberries or sliced strawberries. Herbal tea (chamomile or peppermint).
  **Dinner:** Baked or grilled fish, seasoned with herbs and a dash of olive oil. Mashed potatoes made with skinless potatoes and a little bit of unsalted butter.
  **Environmental, Behavioral, and Social Factors:** The social stigma of a serious chronic illness like CKD may make Ayan feel isolated or reluctant to share his difficulties, though he tries to stay positive and maintain a balanced lifestyle.
  **Assessment:**
  **Anthropometry, Body Composition, and Functional:**
  Weight: Current—75 kg
  Height: 1.71 m
  BMI: 25.7 kg/m^2^
  **Biochemical and Hematological Markers:** creatinine 445.00 mmol/L (increased), elevated eGFR: 14 mL/min/1.73 m^2^, hemoglobin level 82 g/L, PTH level is 90.2 pg/mL. Phosphorus: 1.13–1.78
  **Blood Pressure:** 130/80 mmHg
  **Additional Information:** The disease debuted in March 2022, did not receive treatment, did not asked for help. In August 2023, he began to notice a loss of appetite, nausea and vomiting began to bother him. 21 August 2023 called an ambulance, due to the deterioration of my condition: blood pressure 200/100 mmHg, not reduced by drugs. In this regard, he was taken to the hospital. Taking into account edematous syndrome, shortness of breath, hyperhydration, uremic intoxication, critical indicators of azotemia, anemia, the patient was urgently hospitalized for an emergency hemodialysis session. Since then, he has been receiving hemodialysis courses with repeated hospitalization for inpatient treatment.

## Appendix B. Extended Data Tables and Figures for Methodological Transparency

**Table A1:** Evaluation rubrics using 5-point Likert scale.

Score	Personalization	Consistency	Practicality and Availability
1	Not applicable for evaluation	Not applicable for evaluation	Not applicable for evaluation
2	Poor personalization, addressing a few individual factors like general dietary preferences or habits but without comprehensive tailoring.	Poor consistency, with some recommendations adhering to evidence-based guidelines, but still containing potentially problematic advice or conflicting information.	Poor practicality, with suggestions that may be achievable in some regions or situations, but still contain less accessible or hard-to-find ingredients or foods.
3	Moderate personalization, addressing most individual factors and incorporating them into the recommendations, with improvements possible	Moderate consistency, with a reasonable balance between evidence-based advice and individual tailoring, though improvements can be made to align more closely with established recommendations.	Moderate practicality, with an effort to consider regional availability and ease of implementation, but still with room for improvement to cater to individual context.
4	Good personalization, taking into account a wide range of individual factors such as age, medical history, cultural background, and preferences, with only minor improvements needed.	High consistency, with the majority of recommendations adhering to evidence-based guidelines while demonstrating adaptability to individual needs, with only minor refinements required	Good practicality, with recommendations based on easily obtainable ingredients or foods in the individual’s region, considering cultural habits and familiar meals, with minor refinements needed.
5	Excellent personalization, thoroughly addressing all relevant individual factors, resulting in highly tailored recommendations that cater to specific needs.	Excellent consistency, with all provided recommendations being fully in line with evidence-based guidelines, ensuring safety, efficacy, and tailoring for the specific individual’s needs.	Excellent practicality, with recommendations seamlessly fitting into the individual’s life, ensuring adaptability to their cultural context and basing the suggestions on readily available ingredients or foods

**Table A2:** Summary of Likert-Based Evaluation Frameworks Used in AI Nutrition and Health Studies.

Study	Evaluation Method	Description
Ponzo et al. [23], Nutrients	Likert: Appropriateness, Completeness, Consistency	Evaluated ChatGPT responses against KDIGO and KDOQI guidelines. Responses categorized as “appropriate,” “inappropriate,” “not supported,” “not fully matched,” or “general advice.”
Ponzo et al. [46], JCM	Likert: Accuracy (6-point), Completeness (3-point), Appropriateness, Comprehensibility (3-point)	Used different Likert scales for evaluation, focusing on CKD dietary recommendations.
Kim et al. [33], Frontiers in Nutrition	Likert (0–10): Effectiveness, Balancedness, Comprehensiveness, Flexibility, Applicability, Overall Impression	Evaluated AI-generated vs. control diet plans with professionals in obesity medicine. Additional metrics included personalized diet plan effectiveness, safety, applicability, and likelihood of use. Free-text feedback was also collected.
Naja et al. [59], EJCN	Likert (1–4): Concordance with guidelines, Clarity, Coherence, Practicality	Dietitians evaluated AI chatbot responses for dietary management, nutrition care process (NCP), and menu planning, assessing accuracy and adherence to guidelines. Cohen’s kappa used for inter-rater reliability.
Pugliese et al. [50], Clinical Gastroenterology & Hepatology	Likert: Accuracy, Completeness, Comprehensibility	10 experts in NAFLD and 1 patient advocate rated AI responses using Likert scales, analyzed with descriptive statistics and concordance measures.
Johnson et al. [52], Research Square	Likert (6-point): Accuracy; Likert (3-point): Completeness, Comprehensibility	Physicians from multiple specialties evaluated ChatGPT responses to medical questions based on clarity, completeness, and adherence to guidelines.

**Table A3:** Descriptive nutrient checks for AI-generated meal plans for CKD Stages 1, 2, 4, 5.

CKD Stages	Copilot	Gemini	ChatGPT-4
1	The diet suggested by Copilot is overall well-structured and aligns with patients’ needs during Stage1 of CKD. The generated meal plan provides moderate level of protein where lean protein sources are emphasized. In terms of sodium, avoidance of too much salt and its replacement with herbs and spices are suggested as alternatives which makes the sodium content of the meal plan appropriate for CKD Stage 1 patients. Only concern is pickled vegetables, as they might contain higher amount of sodium. Both potassium and phosphorus levels of the suggested meal plan do not present a risk, as high potassium fruits such as banana and avocado were suggested to avoid. Overall, the plan is appropriate for Stage 1 CKD if portions are controlled and labs are regularly monitored.	The diet suggested by Gemini is well-structured and closely aligns with patient needs as Stage 1 does not present severe restrictions. Protein amount in suggested meal plan is moderate, spread across the meals and protein sources such as lean cuts of chicken, fish and lamb, egg whites, diary products, plant proteins from lentils and nuts were suggested. Sodium content seems to present low risk, as across meal plan, salt was suggested to be replaced with alternatives or with low sodium foods. Potassium and Phosphorus were not restricted, but diet should be monitored as some of the suggested food items might have higher levels of potassium (tomatoes, cucumbers, pumpkin, carrots) and phosphorus (diary products, nuts).	The diet suggested by ChatGPT-4 is well balanced and closely aligns with Stage 1 CKD. Suggested plan provides controlled levels of protein at around 0.8 g per kilogram per day, focusing on the leanest sources and appropriate portions. For Sodium, the risk seems to be minimal—it stays under 2300 milligrams daily, and cooking techniques like boiling meat before stewing to cut down on salt were suggested. For potassium, instead of typical high-K starches, the meal plan uses lower-potassium bases like buckwheat, pumpkin, and berries. For phosphorus is controlled by incorporating grains like buckwheat and quinoa.
2	The diet suggested by Copilot is overall well-structured. The generated meal plan suggests protein sources from moderate sources like fish and lean meat, but it includes Greek Yogurt, which might be high in both protein and phosphorus. Sodium risk is moderate because the plan relies on potentially high-sodium items like canned tuna (even low-sodium varieties) and commercial whole-grain pasta/bread. Potassium is at a moderate risk level due to the inclusion of foods like tomatoes and whole-grain pasta. While it introduces the idea of low-potassium fruits, Greek yogurt and whole might lead to increased levels of potassium and phosphorus.	The diet suggested by Gemini is well-structured and provides appropriate restriction of nutrients. Lean meat and egg whites were suggested as protein sources, while sodium levels were controlled by restricting processed and canned foods. Phosphorus was actively controlled by recommending low-phosphorus milk alternatives and limiting high-phosphorus dairy. However, meal plan includes a small, controlled portion of baked potato, which is considered high-potassium food.	The diet suggested by ChatGPT-4 is well balanced aligns with Stage 2 CKD. Protein restriction is clear and appropriate, restricting it to 0.8 g per kilogram per day. Sodium levels are controlled, with target under 2000 milligrams daily and emphasizing low-sodium yogurt sauces. For both potassium and phosphorus, the plan implements active limitations, strategically using safer, low potassium and low phosphorus grains like quinoa and buckwheat instead of traditional whole-wheat products
4	The diet suggested by Copilot is overall well-structured, with a focus on fresh foods, limited salt and protein intake. Sodium intake is appropriately low due to avoidance of processed foods and salt- based food, which supports kidney protections. The possible concern is potassium and phosphorus level, as in this meal plan high potassium foods are present (tomatoes, nuts, broccoli, carrots), but based on the meal plan the amount is stated reduced, bit not fully clear. Overall, the plan is appropriate for Stage 4 CKD if portions are controlled and labs are regularly monitored.	The diet suggested by Gemini is well-structured and closely aligns with patient needs. Protein restriction is emphasized with a focus on high quality sources such as egg whites, small portions of chicken and fish. Sodium restriction is also strong, with avoidance of processed foods. Phosphorus and potassium are appropriately addressed with limits on dairy, nuts, whole grains, and high potassium fruits and vegetables. Items like spinach, berries, tofu should be used cautiously. The plan also considers coexisting diabetes and anemia, ensuring carbohydrate control and iron support. Overall, the plan is appropriate for Stage 4 CKD, but close monitoring of labs is needed.	The diet suggested by ChatGPT-4 is well balanced and closely aligns with Stage 4 CKD with coexisting type 2 diabetes. Protein restriction is clear and appropriate, with high quality sources (egg, chicken, fish) in limited portions. Sodium control is strong, with target under 1500 mg/d. Potassium and phosphorus are managed effectively by avoiding high-potassium and high phosphorus foods. Fruits are limited to low potassium options. The plan also considers coexisting diabetes and anemia by adding low glycemic index grains and iron rich foods. Overall, this diet is appropriate for Stage 4 CKD with diabetes with a clear portion sizes and considering as well cultural preferences and alternatives.
5	This plan shows nutrient balance, cultural adaptation and key priorities. Protein intake is appropriately increased, with lean sources like chicken, fish and egg whites. Sodium restriction is noted, but ideally it should be <2000 mg/day, with strict avoidance of processed foods, canned foods and added salt. Potassium and phosphorus manaGeminient needs closer adjustment. Some foods (potatoes, broccoli, nuts, cottage cheese, yogurt) are high in potassium or phosphorus and need portion control. Spinach should be avoided due to high potassium/phosphorus. Overall, the diet is appropriate for CKD Stage 5 patients on dialysis but would be safer with stricter sodium limits and careful adjustment of potassium and phosphorus sources.	The plan is well structured and correctly increases high quality protein for dialysis patients. Sodium and fluid control must be strict, < 2000 mg sodium/day. Phosphorus control remains a concern, as items like cottage cheese, nuts, yogurt are included but may raise phosphorus. Potassium manaGeminient is addressed with low potassium fruits and vegetables, but strict portion control is needed to stay safe. Overall, suggested plan is appropriate for a dialysis patient but needs tighter control on potassium and phosphorus.	This plan is well prepared for a dialysis patient: protein intake increased, potassium and phosphorus control are addressed with substitutions (avoiding tomato, turnips instead potatoes). Overall, this diet is appropriate, but close monitoring of phosphorus (hummus, dairy, flatbread) and potassium (fruit portions) remains essential.

**Table A4:** Key dietary recommendations for CKD stages, guidelines used in AI Diet Assessment.

CKD Stage	Recommendations	Sources
1–2	- Protein Intake: 0.8–1.0 g/kg/day. - Sodium Intake: Limit to less than 2300 mg/day. - Potassium and Phosphorus: Generally unrestricted unless serum levels are elevated. - Calories: Ensure adequate caloric intake to support energy needs. - Dietary Advice: Promote a balanced diet rich in fruits, vegetables, and whole grains.	- KDOQI Guidelines (2020) [41] - National Institute of Diabetes and Digestive and Kidney Diseases (NIDDK) [42]
3–5 (Non-Dialysis)	- Protein Intake: 0.6–0.8 g/kg/day (some guidelines recommend as low as 0.55–0.60 g/kg/day). - Sodium Intake: Limit to less than 2300 mg/day. - Potassium: Adjust intake based on individual lab results; restrict if hyperkalemia is present. - Phosphorus: Limit to 800–1000 mg/day; avoid phosphorus additives. - Calories: 30–35 kcal/kg/day to maintain energy balance. - Fluid Intake: Adjust based on medical status.	- KDOQI Guidelines (2020) [41] - ESPEN Guidelines [43] - European Renal Best Practice (ERBP) [60] - UK Kidney Association [61]
4–5 (Pre-Dialysis)	- Protein Intake: 0.6–0.8 g/kg/day (some guidelines recommend up to 1.0 g/kg/day). - Sodium Intake: Limit as per blood pressure and fluid status. - Potassium and Phosphorus: Restrict intake; monitor serum levels closely. - Calories: 30–35 kcal/kg/day. - Fluid Restriction: Implement if edema develops. - Micronutrients: Ensure adequate intake of vitamins and minerals; supplement as needed.	- Clinical Guideline of the Republic of Kyrgyzstan - KDOQI Guidelines (2020) [41] - NICE Guideline [NG203] (2021) [44]
Dialysis	- Protein Intake: - Hemodialysis: 1.1–1.4 g/kg/day (some guidelines recommend up to 1.5 g/kg/day). - Peritoneal Dialysis: 1.0–1.2 g/kg/day. - Calories: 30–40 kcal/kg/day depending on age and physical activity. - Sodium, Potassium, Phosphorus: Monitor and adjust intake as necessary. - Fluid Intake: Adjust based on urine output and fluid gains during dialysis. - Micronutrients: Supplement water-soluble vitamins.	- ESPEN Guidelines [43] - Clinical Guideline of the Republic of Kyrgyzstan - UK Kidney Association (UKKA) [61] - National Kidney Foundation (NKF) [2]
General Recommendations	- Limit Salt Intake - Control Protein Intake - Heart-Healthy Diet - Limit Phosphorus and Potassium: - Avoid Certain Foods	- Cleveland Clinic [62] -Physicians Committee for Responsible Medicine [63] - National Institute of Diabetes and Digestive and Kidney Diseases (NIDDK) [42] - Clinical Guideline of the Ministry of Health of Kazakhstan

**Table A5:** Effect Sizes (Cliff’s δ) for GPT, Copilot, and Gemini Across Personalization, Consistency, and Practicality.

	Personalization		Consistency		Practicality	
Comparison	Cliff’s Delta (δ)	95%CIs	Cliff’s Delta (δ)	95%CIs	Cliff’s Delta (δ)	95%CIs
GPT–Gemini	0.20	[0.04; 0.35]	0.18	[−0.01; 0.34]	0.20	[0.02; 0.37]
GPT–Copilot	−0.27	[−0.45; −0.06]	−0.24	[−0.43; −0.03]	0	[−0.20; 0.20]
Gemini–Copilot	−0.47	[−0.62; −0.28]	−0.42	[−0.59; −0.22]	−0.20	[−0.37; −0.02]

**Table A6:** Inter-Rater Reliability of Expert Evaluations Using Krippendorff’s Alpha.

Evaluation Criterion	Krippendorff’s Alpha	Interpretation
Personalization	0.22	Fair Agreement
Consistency	0.11	Slight Agreement
Practicality	−0.2	Poor/No Agreement

**Table A7:** Mean Absolute Percentage Error and 95% Confidence Intervals for Nutrient Estimates.

Nutrient	Mean Absolute % Error	95% CI (Lower–Upper)
Protein	28.22%	−9.59–66.03%
Sodium	52.01%	15.14–88.89%
Potassium	53.67%	24.31–83.02%
Phosphorus	31.86%	14.16–49.56%

**Table A8:** Qualitative analysis of AI-generated meal plans.

CKD Stages	AI Model	Suggested Food	Issue
1	Copilot	Brown rice	Rarely consumed in the local diet and not part of routine food culture
	Gemini	Brown rice	Rarely consumed in the local diet and not part of routine food culture
	ChatGPT-4	Brown rice	Rarely consumed in the local diet and not part of routine food culture
2	Copilot	Grilled fish like salmon or trout, marinara sauce, almond milk	Rarely consumed in the local diet and not part of routine food culture; limited availability and higher cost
	Gemini	Low-phosphorus milk alternative, low-phosphorus cream cheese, brown rice, unsalted rice cakes,	Rarely consumed in the local diet and not part of routine food culture; limited availability and higher cost
	ChatGPT-4	Almond milk, yogurt-based garlic sauce	Rarely consumed in the local diet and not part of routine food culture; limited availability and higher cost
3	Copilot	Quinoa, tofu, brown rice,	Rarely consumed in the local diet and not part of routine food culture; limited availability and higher cost
	Gemini	Low-phosphorus milk alternative, quinoa, tofu, brown rice, unsalted rice cakes	Rarely consumed in the local diet and not part of routine food culture; limited availability and higher cost
	ChatGPT-4	Quinoa	Rarely consumed in the local diet and not part of routine food culture
4	Copilot	Hummus	Rarely consumed in the local diet and not part of routine food culture
	Gemini	Tofu, rice cakes (unsalted)	Rarely consumed in the local diet and not part of routine food culture
	ChatGPT-4	Barley tea, low-sodium hummus, cod or tilapia	Rarely consumed in the local diet and not part of routine food culture; limited availability
5	Copilot	Peppermint tea	Rarely consumed in the local diet and not part of routine food culture
	Gemini	Low phosphorus milk alternative, rice cakes (unsalted)	Rarely consumed in the local diet and not part of routine food culture; limited availability and higher cost
	ChatGPT-4	Hummus, cod or tilapia	Rarely consumed in the local diet and not part of routine food culture, higher cost
